# Strengthening the role of Community Health Representatives in the Navajo Nation

**DOI:** 10.1186/s12889-017-4263-2

**Published:** 2017-04-21

**Authors:** Caroline King, Alex Goldman, Vikas Gampa, Casey Smith, Olivia Muskett, Christian Brown, Jamy Malone, Hannah Sehn, Cameron Curley, Mae-Gilene Begay, Adrianne Katrina Nelson, Sonya Sunhi Shin

**Affiliations:** 1 Division of Global Health Equity, Brigham and Women’s Hospital, 75 Francis Street, Boston, MA 02115 USA; 2 Community Outreach and Patient Empowerment (COPE), Gallup, NM USA; 3 Tufts University School of Medicine, Boston, MA USA; 4 Harvard Medical School, Boston, MA USA; 5 Partners In Health, Boston, MA USA; 6 Navajo Nation Community Health Representative Outreach Program, Window Rock, AZ USA; 7 Navajo Nation Department of Health, Window Rock, AZ USA; 8 Department of Global Health and Social Medicine, Harvard Medical School, Boston, USA

**Keywords:** Community health workers, Community health representatives, American Indians, Electronic health records, Diabetes, Chronic disease, Clinic-community linkages

## Abstract

**Background:**

Strengthening Community Health Worker systems has been recognized to improve access to chronic disease prevention and management efforts in low-resource communities. The Community Outreach and Patient Empowerment (COPE) Program is a Native non-profit organization with formal partnerships with both the Navajo Nation Community Health Representative (CHR) Program and the clinical facilities serving the Navajo Nation. COPE works to better integrate CHRs into the local health care system through training, strengthening care coordination, and a standardized culturally appropriate suite of health promotion materials for CHRs to deliver to high-risk individuals in their homes.

**Methods:**

The objective of this mixed methods, cross sectional evaluation of a longitudinal cohort study was to explore how the COPE Program has effected CHR teams over the past 6 years. COPE staff surveyed CHRs in concurrent years (2014 and 2015) about their perceptions of and experience working with COPE, including potential effects COPE may have had on communication among patients, CHRs, and hospital-based providers. COPE staff also conducted focus groups with all eight Navajo Nation CHR teams.

**Results:**

CHRs and other stakeholders who viewed our results agree that COPE has improved clinic-community linkages, primarily through strengthened collaborations between Public Health Nurses and CHRs, and access to the Electronic Health Records. CHRs perceived that COPE’s programmatic support has strengthened their validity and reputation with providers and clients, and has enhanced their ability to positively effect health outcomes among their clients. CHRs report an improved ability to deliver health coaching to their clients. Survey results show that 80.2% of CHRs feel strongly positive that COPE trainings are useful, while 44.6% of CHRs felt that communication and teamwork had improved because of COPE.

**Conclusions:**

These findings suggest that CHRs have experienced positive benefits from COPE through training. COPE may provide a useful programmatic model on how best to support other Community Health Workers through strengthening clinic-community linkages, standardizing competencies and training support, and structuring home-based interventions for high-risk individuals.

## Background

The Navajo Nation, located in the Southwestern United States and encompassing parts of Utah, New Mexico and Arizona, is the second largest Native American tribe in the United States with a population of 332,000 people in 2010 [1]. The Navajo Nation is actively seeking ways to help address the high prevalence of Type 2 Diabetes Mellitus (DM) on the reservation, which in 2008 was 21.47% in people over 20 years old [2]. This challenge is compounded by the distance needed to travel to purchase food either on or off the reservation. Spread out over 27,000 mile^2^, the Navajo Nation is the largest tribe by landmass in the United States [3].

The Navajo Area Indian Health Services is one of 12 geographic areas of the Indian Health Service, an agency of the US Public Health Service Department of Human Health Services. Navajo Area Indian Health Services are provided by 5 hospitals, 7 health centers and 15 clinics throughout the region [4]. While some of these facilities are operated by Indian Health Services, additional facilities are run by the Navajo Nation through 638, contract, or compact agreements [4].

### Navajo nation Community Health Representatives (CHRs)

Globally, Community Health workers (CHWs) have been shown to play a vital role in improving the health of communities [5–10]. Today, the Navajo Nation employs and pays 118 CHRs (Navajo CHWs) who work across the reservation. Started in 1968 as a partnership with tribal leaders, the CHR program has delivered culturally-appropriate healthcare information and services to Native communities [1]. They collectively treat roughly 8000 clients per year [11, 12]. CHRs must be fluent in both Navajo and English, and must be trained as a Certified Nursing Assistant. CHRs are trained to handle a variety of health care issues, though typical topics addressed may vary based on the types of referrals CHRs receive from the hospital in their Service Unit. CHRs fulfill a role critically important to health on the Navajo Nation. As members of the communities they work in, CHRs have roots in the communities they serve. This community connection is reinforced through CHR home visits with clients, providing an opportunity for service delivery in a fashion that works concurrently with both western and traditionally Native healthcare systems to support the health of community members.

The Navajo CHR Program has recently also begun to enroll CHRs into a Public Health Certificate course dually awarded by Dine College and Arizona State University, who oversees the certification process. CHRs are organized as teams corresponding to each Service Unit; each team is managed by a Supervisor. Each Navajo Nation service unit typically have between 2 and 15 CHRs on their team, depending on the size of the service unit. The Navajo Nation service units are further divided into smaller chapters. There is one CHR assigned to each chapter. The number of CHRs in each service unit reflects the number of chapters within the service unit, though vacancies may also influence this number. Office Assistants typically work at each CHR Service Unit office to support CHR teams and clients, fulfilling tasks including answer phones and supporting team activities. Teams meet and work together at functions within their Service Unit as decided by their Supervisor, including health fairs, school health screenings and health education trainings. CHRs usually work in both offices outside of health facilities in their Service Units as well as in their local home communities.

Before COPE began working with the CHR program, CHRs did not have access to Electronic Health Records (EHR) for patients; this was in part due to logistic, training, data accessibility, and data sharing issues. CHR communication directly with physicians providing care was and is often limited, particularly in Service Units without EHR, where there is often no way for CHRs and most physicians to connect other than phone calls from the field to a physician’s office. High provider turnover has contributed to a lack of awareness of CHRs among providers at some sites.

### COPE intervention

Despite the long-standing presence of CHRs in the community, efforts to build clinic-community linkages between CHRs and clinical teams have been limited. The Community Outreach and Patient Empowerment Program (COPE) is a Native non-profit organization with formal partnerships with both the Navajo CHR Program and the clinical facilities in the region, implemented through partnership agreements. COPE works to better integrate CHRs into the local health care system through training, system improvements in care coordination, and a standardized suite of health promotion materials for CHRs to deliver to high-risk individuals in their homes [13].

COPE began in 2009 in partnership with Navajo Nation. As a Partners in Health site, COPE applies the Partners in Health global model of building capacity and improving health outcomes, primarily through heavy reliance on CHWs [14]. COPE is further aligned with Partners in Health’s approach of “public sector accompaniment,” by, focusing efforts on strengthening the public sector system of health services rather than creating alternative or complementary programs.

For individuals living with uncontrolled DM, COPE has developed a flexible curriculum of “modules”; CHRs are provided modules in printed format as flipcharts or electronically on pre-configured tablets in order to provide health promotion and coaching to their high-risk clients. Patients are enrolled into the COPE program either by the CHR or via provider referral. As a programmatic (not research) service, written informed consent is not required; however patients are asked if they are willing to participate in COPE, involving regular home visits for health education teaching. For those who agree to participate, CHRs typically visit patients once or twice a month, and review a module during most but not all visits. Each teaching module is structured using a motivational interviewing approach.

Though the COPE intervention was intended to be focused on patients with DM, and particularly those with uncontrolled DM, CHRs have flexibility in who they choose to enroll in the COPE Program, which often is based on CHRs perceptions in the field of who may benefit. We sought to clarify enrollment strategies in our qualitative analysis included in this paper.

Public Health Nurses (PHNs) had been serving in a liaison role between CHRs and clinical teams prior to the implementation of COPE. They continue to accomplish this through joint home visits, case management, and phone calls, though there is variability of this coordination across sites. Some physicians connect with CHRs at monthly case management meetings organized by COPE, held among CHRs, the CHR supervisor, and local physicians interested in participating.

Monthly training sessions at each service unit are coordinated by COPE and taught by local providers to build CHR-provider relationships. Teaching materials, learning objectives, and competency assessments are developed in advance with clinician feedback for each health topic. COPE also provides training to CHRs and their supervisors in Motivational Interviewing, self-care and wellness, and team-building.

COPE has worked extensively to better integrate CHRs into clinic-based care teams. In addition to involving providers in training and curriculum development, COPE has established referral processes in order for providers to refer patients to CHRs, as well as case management meetings that involve both CHRs and hospital providers at Service Units. COPE has also worked to improve the awareness of the CHR program among providers by presenting to hospital staff about CHR work. COPE has supported joint home visits between providers and CHRs and has enabled CHR access to the EHR in several Service Units.

The objective of this mixed-methods study was to understand how CHRs perceived COPE’s role in CHR work among each of the eight CHR teams in the Navajo Nation. COPE sought to address this study objective through cross-sectional surveys of CHR teams and through CHR focus group interviews.

## Methods

### Study design

This study was designed as a component convergence mixed-methods study, during which CHR surveys and CHR focus groups occurred separately and concurrently. Data mixing occurred in the interpretation phase [15]. Results from both quantitative and qualitative data were considered of equal importance to the study question. In using this convergence design, overlapping themes of inquiry between survey and focus group data were used for triangulation (e.g. communication with clinical staff, access to EHR). Expansion techniques were used with qualitative data to further understand issues brought up with quantitative data (e.g. reported success of trainings were due to access to useful educational materials) [15]. Surveys were completed during the summers of 2014 and 2015; focus groups were completed from November 2014 through January 2015.

### Participants

A total of 107 CHRs responded to the CHR survey. Nearly all of the CHRs in Navajo at the time of surveys and focus groups were female. Roughly 53 of the 85 CHRs responded to the 2014 survey and roughly 54 of the 90 CHRs responded to the 2015 survey, representing approximately 62.4% and 60% of the total staff respectively. Thus, the population surveyed in 2014 and 2015 were both cross-sectional in design, as were the focus groups, to understand the ongoing perception of COPE in this longitudinal cohort study.

### Data collection

From November 2014 to January 2015, eight focus groups (comprised of CHRs and occasionally Office Assistants at each service unit) were conducted using semi-structured interview guides developed by COPE staff with input from both advisory groups. The eight focus group interviews included a total of 53 of the roughly 90 CHRs and two of the roughly 8 Office Assistants, representing approximately 56.1% of the total CHR staff. Interviews focused on CHRs’ views on their own work and COPE’s influence on their program. Focus groups were conducted in English by researchers trained in qualitative methods, although participants did occasionally switch to Navajo during interviews. There were three instances when focus group participants switched to Navajo, and the research team deemed it too disruptive to ask for translation from Navajo to English at the time of the interview, and so Navajo comments were excluded from transcripts. COPE’s Community Outreach Specialist provided feedback that participants may be switching to Navajo to share a thought not easily expressed in English. Focus groups were unable to be recorded in Navajo, and so study team members typed a transcript of interviews during the focus groups after receiving verbal consent from participants to do so. Interview notes were incorporated into the transcription and finalized upon consensus by all note takers. All data were de-identified.

Additionally, COPE established two stakeholder advisory bodies: the COPE Advisory Group (CAG) in 2012 and the Community Health Advisory Panel (CHAP) in 2013. Each group meets 3 to 4 times a year to provide oversight and input on COPE’s services and research. CAG is comprised of local physicians, nurses, program leaders, information technology specialists, Navajo Nation Department of Health program directors and CHR supervisors. CHAP is composed of patients, patient relatives, and CHRs. CHAP and CAG both provided input on the design and interpretation of this study. CHAP and CAG provided input into themes and language to include in our surveys and focus group interviewing guides (Table 1). CHAP also provided feedback on our CHR survey (described below) before it was utilized. Finally, CHAP and CAG both reviewed our qualitative and quantitative results to provide feedback on our interpretation, which has been included throughout this manuscript.

**Table 1:** CHR focus group interview guide

Background Questions	Tell me a little bit about how you became a CHR? (Probe: what were your thoughts about the CHR program before you started?)What motivated you to be a part of the CHR program?
	What past experience has helped with your work with the CHR Program?
	Tell me how your ability to speak Navajo has changed since you started working for the CHR Program? Has that changed the way you connect with your clients?
Work/Actual Home Visits/Interactions with patients	Tell me about a typical home visit? (Probe: What things do you do other than health related activities? Do you use COPE materials?)
	How do you explain COPE to a client?
	How do you choose which clients to enroll into COPE?
	Tell me about a time when you felt that you really met the needs of your client? How about a time where it was more difficult to meet their needs?
	Is a home visit to COPE patient different from home visit to non-COPE patient? (Probe: If so, how? If not, what how are they similar?)
	Have COPE training sessions changed your practice? (if yes, why + how? if not, why not?)
Beliefs + attitudes about role in community/health/family	What does it mean to be a CHR? (Probe: what do you feel you do beyond your requirements as a CHR?)
	What support does a CHR need in order to be effective?
	What is “healthy” mean to you? Your clients? (Probe: traditional? spiritual? community?) How does that impact your practice?
	Share an example of your interactions with a client’s family?

As mentioned above, a CHR survey was developed by COPE staff in 2014 with the overall objective of determining COPE’s effect on CHR activities, including patient interaction and communication between CHRs and providers (Table 2). Questions for this survey were created based on feedback from our CAG and CHAP groups as to what aspects of CHR patient care were most important to understand. Themes used in survey questions were designed from discussions among CHAP and CAG participants. When Likert scaling from 1 to 10 was used, responses were classified as 1–3 = strongly negative, 4–7 = neither strongly positive nor strongly negative, and 8–10 = strongly positive. This paper survey was administered to CHRs and CHR supervisors after a monthly session at all eight Service Units in the summer of 2014 and again in the summer of 2015. A COPE staff member was present to distribute and collect surveys and to answer any questions. A data entry form and codebook were created using EpiData 3.1.

**Table 2:** CHR survey questions

CHR Case Load	How many chapters do you currently cover?
	How many clients do you have in each of these categories: COPE clients? Non-COPE clients? High Risk clients?
	How many years have you worked as a CHR?
Services Provided	How many home visits a month do you make to COPE Clients? Non-COPE clients? High Risk Clients?
	How often do you provide health education to your clients?
	How often do you work with your client to set goals?
	How often do you check your client’s blood pressure?
	How often do you check your client’s blood sugar?
	During an average home visit to a client, how long do you spend providing health education?
	During an average home visit to a client, how many health educations do you provide?
Training and Team Communication	How useful are COPE Motivational Interviewing trainings for you?
	How useful are COPE monthly Health Trainings (flipchart trainings?)
	Do you feel that you receive enough training for your work as a CHR?
	How do you feel about the amount of time you spend in COPE training each month?
	How easy is it for you to get a hold of a provider about a client?
	How easy is it for you to get a hold of a PHN about a client?
	Do you feel comfortable speaking Navajo with your clients?
Perception of COPE	Because of COPE, are you able to help clients make healthy changes in their diet?
	Because of COPE, are you able to help clients make health changes in exercise?
	Because of COPE, are you able to help clients make health changes in taking their medications?
	Because of COPE, are you able to help clients make healthy changes in seeing providers more regularly (like the doctor, nutritionist, or DM Educator)?
	Do you think COPE is helping to improve the referral process from provider to CHRs?
	Do you think COPE is helping to improve client communication between CHRs and providers (including nurses, providers, other specialists)?
	Do you feel that COPE is helping CHRs and PHNs work more effectively together?
	Because of COPE, do you think that CHRs help patients lower their A1C?
	Because of COPE, do you think that CHRs help patients lower their blood pressure?
	If your service unit holds COPE case management meetings, are these helpful for you?
	If your service unit does not hold case management meetings, would you like case management meetings in your service unit?

### Data analysis

Qualitative interviews were then coded and analyzed. Focus groups were analyzed using a thematic analysis via an inductive process using Dedoose. From this, two themes of COPE’s effect were identified: Perceptions of COPE’s role in clinic-community linkages and CHR views on their client interactions.

Surveys were coded by hand and then double entered into EpiData. Results of the CHR survey were analyzed by both year and service unit. Arithmetic mean is used to present descriptive statistics for variables from the survey because of the relative normality of the data based on sample size. Pearson’s chi-squared test was used to evaluate associations between pre-specified categorical variables related to CHR feelings about their workload. Surveys were anonymous; therefore individual respondents could not be tracked from 2014 to 2015.

The study was approved by the Ethics Committee of Partners Healthcare and the Navajo Nation Human Research Review Board.

## Results

Results from focus groups and surveys overlap and suggest that CHRs value COPE training sessions and use COPE training materials in the field with clients. Furthermore, the experience of CHRs working with clinical teams and accessing EHR systems varies by Service Unit in terms of the number of referrals CHRs receive, how CHRs divide their time, and how CHRs interact with hospital systems. Our qualitative findings provided greater insight into why CHRs valued training session and how they implemented COPE materials in the home (Table 3). Furthermore, focus group results revealed that the integration of CHRs with clinical teams was varied from service unit to service unit, with greatest integration perceived at sites where CHRs had gained access to the EHR.

**Table 3:** Key qualitative domains and themes

Qualitative domains	Key qualitative themes
Clinic-Community Linkages	CHRs perceive that COPE has helped: • Strengthen PHN-CHR alliances • Introduce case management to Service Units • Create CHR access to the HER • Improved bonds among CHR teams • Supported communication and coordination of care in Service Units
Client Interactions	• CHRs enroll COPE clients in many different ways • Most, but not all, COPE clients were individuals with DM • Individual decisions by CHRs related to enrolling a patient in COPE included whether or not a patient had uncontrolled DM, how long they had DM, their blood sugar and/or A1c at one or several visits, and their vital signs

### CHRs workload

CHRs reported a wide variety of experience, ranging from trainees who were attending their first COPE session to those who had been working in the field for over 20 years (Table 4). The average experience as a CHR was 10 years. The average number of chapters covered was 1.84 with a range of 0–4. The CHR caseload consisted of COPE clients (mean = 5.83), non-COPE clients (mean = 52.86), and high risk clients that could be classified as COPE or non-COPE (mean = 13.63). There were no significant differences in any of these variables by year (one way t-test, *p* > 0.05). There were expected differences in numbers of clients in each service unit.

**Table 4:** Summary of CHR survey demographics, experience and client load

	Frequency	Percent-age	2014 frequency	Percent-age	2015 frequency	Percent-age	Average years of experi-ence	Average chapters covered	Client load: COPE	Client load: non-COPE	Client load: high risk
Chinle	17	15.89	8	15	9	16.67	8.6	1.76	4.33^a^	39.88^a^	12.38
Crownpoint	14	13.08	6	11.3	8	14.81	14.4^a^	2	7.09	74.11^a^	13.50
Ft. Defiance	13	12.15	7	13.2	6	11.11	6.8^a^	1.54^a^	6.50	89.60^a^	10.23^a^
Gallup	17	15.89	7	13.2	10	18.52	8.2	2.12^a^	7.30^a^	26.67^a^	14.40
Keyenta	9	8.41	4	7.5	5	9.26	13.0^a^	1.56^a^	4.625	23^a^	16.78^a^
Shiprock	23	21.50	11	20.8	12	22.22	11.4	1.79	0.89^a^	54.82	6.14^a^
Tuba City	7	6.54	5	9.4	2	3.70	11	3^a^	1.75^a^	31.75^a^	40.00^a^
Winslow	7	6.54	5	9.4	2	3.70	7.5^a^	1.14^a^	13^a^	29.43^a^	18.56^a^
Total	107	100	53	100	54	100	10	1.85	5.93	54.41	13.58

^a^Indicates a response significantly different from the grouped mean (T-test, *p* < 0.05)

### Clinic-community linkages

When surveyed, 44.6% of CHRs felt strongly that communication and teamwork had improved; 40.59% felt that the patient referral process from providers to CHRs have improved. In the qualitative interviews, CHRs note improved collaboration with Public Health Nurses (PHNs) because of connections through COPE. In some Service Units, this collaboration was created through case management meetings, where CHRs and PHNs come together to discuss mutual patients. In others, COPE would coordinate meetings at sites that brought together PHNs, CHRs, and other health professionals. These meetings were usually focused on creating a robust referral system for clients to connect to CHRs, though “meet and greet” type meetings have also been held. Not all CHRs would attend “meet and greets” or referral system meetings, but often the presence of any CHRs, or the CHR supervisor, helped to establish a link between the CHR office and PHNs.

In focus groups, CHRs reported that communication with clinic providers had improved, citing improvements in the number client referrals, the reliability of the referral system and the effect of holding case management meetings. CHRs particularly enjoyed traveling with providers into the field to visit clients and felt that such visits helped bolster their credibility with clients. CHRs also appreciated that providers led COPE trainings and felt that this helped with provider familiarity:

“What I like is that they [COPE] bring some of the providers over from next door to be some of the teachers.”

- CHR, Focus Group 4

CHRs consistently expressed their desire and perceived importance in working more closely with physicians and nurse practitioners. While CHRs described continued challenges in achieving these linkages, they acknowledged that incremental improvements in communication that had come about due to the COPE Program. A notable change in some service units was gaining access to the EHR. CHRs from service units with access to EHR felt the ability to document their client encounters improved communications between providers and CHRs. Furthermore, providers were able to recognize CHRs as part of their clients’ care team.

“I think we have better communication with the doctors, now with the EHR. I met a lot more doctors, they say, ‘you’re so and so,’ and we email each other.”

- CHR, Focus Group 1

The improved communication through the EHR was bi-directional. CHRs appreciated gaining access to client information, which they felt had direct implications on their ability to improve the health of their clients. For many teams, however, these improvements in clinic-community linkages remained sporadic or indirect. For instance, CHRs in one Service Unit noted that they were able to call physicians directly to discuss clients, but this was often specific to “champion providers” who were very receptive to CHRs and not the norm. Some CHRs felt they had better access to providers through PHNs, reflecting their improved communication with PHNs but a continued lack of direct communication with providers. In fact, some teams felt that PHNs were still the only providers they could collaborate with.

“We have not had more contact with physicians, but [have had] more interaction with PHNs.”

- CHR, Focus Group 2

CHRs who had access to the EHR reported greater ability to contact providers about patient issues (chi-square, *p* < 0.05). EHR access did not appear to effect COPE’s ability to affect change in PHN/CHR communication, client communication between CHRs and providers, or CHRs and PHNs working more effectively together (chi-square, *p* > 0.05). There were no clinically relevant differences between service units or years when CHRs were asked about communication between CHRs and providers or CHRs and PHNs.

CHRs also felt that collaboration among CHRs within each service unit was positively effected by COPE. They felt that COPE helped to “build morale” and “put teams on the same page.” Because of the rural nature of the Navajo Nation, CHRs are often isolated from one another during their day-to-day work, and may only spend time altogether a few times a month. Monthly COPE trainings created a time for CHRs to “come together and regroup.” CHRs reported the desire to spend more time together outside of COPE trainings. They also noted that when they are out in the field, knowing that other CHRs are using the same materials from their COPE trainings creates a shared sense of education, training, and purpose, and may help them feel more connected to other CHRs.

“We all use all the info we get from COPE, [and] that shows we work as a team.”

- CHR, Focus Group 4

It is important to note that many CHRs felt that their teams collaborated well before COPE began, but that COPE enhanced this closeness of CHR teams and their sense of belonging to “one family.” One participant felt paperwork and an emphasis “on the numbers” may take away from time CHRs would otherwise spend together.

There was variability regarding how COPE influenced the relationship between CHRs and their supervisors. Some CHRs felt that their supervisors were looking out for them because they had brought in COPE to lead trainings. Others felt that COPE supported supervisors in being stronger advocates for the CHR Program with other stakeholders.

“I think [collaboration] has changed. Before we didn’t have [] supervisor, once we got a supervisor our supervisor was collaborating with outside resources such as COPE and the hospital.”

- CHR, Focus Group 2

In terms of building linkages between CHRs and other community stakeholders, collaboration between CHRs and local chapter houses may have mildly improved through the group teachings at local community meetings using COPE materials, which were well-received by the community. In terms of other programs involved in community outreach aside from PHNs (e.g. senior centers, Navajo Nation Special Diabetes Program), CHRs generally did not perceive closer collaboration with these groups through COPE.

“Sometimes [at the chapter houses] I do Healthy Heart training. During the summer I did a lot of healthy cooking from the Healthy Heart training…. It helped me develop a class.”

- CHR, Focus Group 2

Overall, CHRs identified COPE’s role in strengthening PHN-CHR alliances, introducing case management, creating CHR access to the EHR, and strengthening bonds among CHR teams as having the greatest effect on improved communication and coordination of care.

### Client interactions

In terms of COPE enrollment, CHRs reported enrolling COPE clients in a variety of ways. Most COPE clients were individuals with DM. The process by which CHRs selected clients for COPE enrollment varied. Some CHRs actually enrolled all of their high-risk or elderly clients into COPE. Other CHRs did not enroll their entire high-risk or elderly list in COPE but, instead, chose clients on a case-by-case basis. In these cases, CHRs reported choosing clients for COPE based on a variety of criteria: whether or not they had uncontrolled DM, how long they had DM, their blood sugar and/or A1c at one or several visits, and their vital signs.

Provider referrals for both COPE and non-COPE patients also varied by Service Unit. Some CHRs only received referrals for COPE patients from providers for clients with uncontrolled DM or complications related to it. Other Service Units referred a variety of different clients, for both COPE and non-COPE referrals, including post-partum clients or clients who needed immunizations.

Additionally, given CHR turnover, CHRs also took over existing COPE clients when a CHR retired, resigned, or transferred. When asked whether clients left the COPE program because they had completed the curriculum or asked to be removed, CHRs agreed that they rarely removed clients off COPE curriculum.

In terms of services delivered, CHRs acknowledged that COPE has broadly influenced how they work with their clients. CHRs uniformly reported that they used the COPE flipcharts with all of their clients, not just with high-risk clients. Most CHRs reported that home visits using the curriculum COPE provided were not longer than visits before COPE started, however a few felt that the visits are now slightly longer.

When surveyed, CHRs felt strongly that COPE’s trainings were useful, with 80.20% of CHRs responding strongly positively. During the qualitative interviews, CHRs highlighted the COPE flipcharts and said that they improved their credibility with their clients, and that clients, especially elders, really liked the emphasis on visual teaching points.

“We get a lot of credit when we use the flipcharts. It helps us more, and the people are really enjoying the flipcharts. They’re like, ‘Oh, you have this in writing. You are not just talking.”

- CHR, Focus Group 3

CHRs surveyed found motivational interview training useful with 64.36% responding strongly positively. When asked in the qualitative interview, CHRs felt that their clients appreciated when they used Motivational Interviewing techniques during home visits, and that Motivational Interviewing increased trust between CHRs and clients. In COPE’s Motivational Interviewing curriculum, CHRs are encouraged to explore how a client is feeling about a given topic, rather than offering direct advice without being asked. Some CHRs reported spending entire home visits just listening to clients express their emotions.

“[The Motivational Interviewing training] taught us how to communicate with our clients, how to talk to them, to not just give [our clients] yes and no questions. Just kind of [how to] get them to talk to us more.”

- CHR, Focus Group 1

CHRs also noted that Motivational Interviewing was appropriate for some encounters and not for others. At times, CHRs felt that it was important to be more direct and honest with their clients about the seriousness of their conditions; it really “opened their [client’] eyes” to what they needed to do to be healthy.

**Fig. 1:**
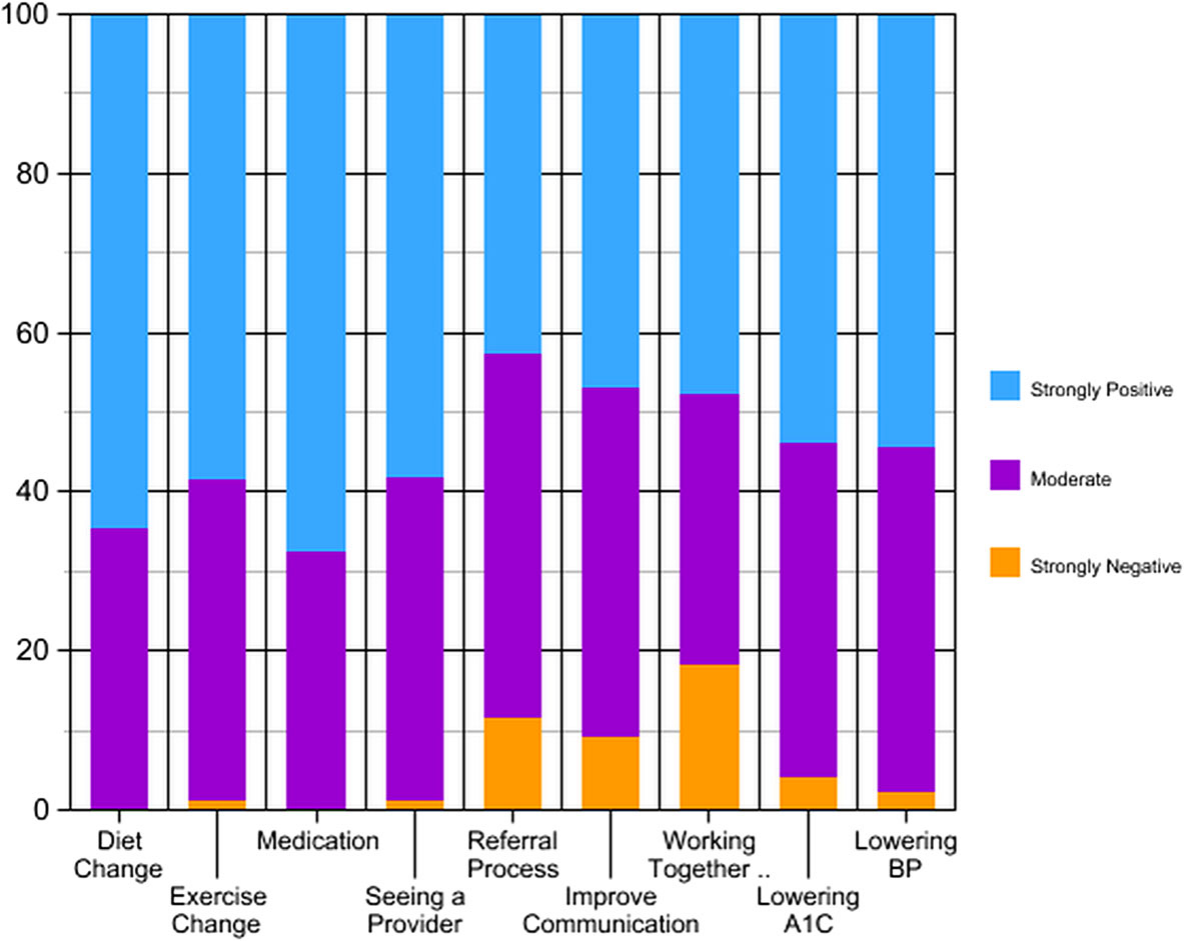
CHRs perception of COPE in effecting CHRs ability to support clients and communicate with healthcare teams

Finally, as shown in Fig. 1, when asked if CHRs felt that COPE has improved the health of their patients, most CHRs strongly agreed that patient behavior had improved, including changes in diet, exercise, medication adherence and provider follow-up. CHRs felt that COPE resulted in improvement on clinical measures such as A1C (51.46%) and blood pressure (52.43%).

## Discussion

Programmatic efforts to strengthening Community Health Worker programs have been recognized to increase access to chronic disease prevention and management efforts in low-resource communities. Since the beginning of the COPE Program’s work in the Navajo Nation in 2009, COPE has sought to strengthen the opportunities CHRs have to improve the health and well-being of their clients.

Stakeholders, including CHRs who viewed our results as a part of CHAP, agree that COPE has improved clinic-community linkages, primarily through strengthened collaborations between PHNs and CHRs as well as access to the EHR. Although limited, there have also been improvements in communication between clinic-based providers (e.g. physicians, Nurse Practitioners, etc.) and CHRs. An interesting finding has been the role that COPE has played in strengthening internal bonds within CHR teams, along with the expressed desire for COPE to continue their efforts to strengthen CHR-provider, CHR-CHR and CHR-supervisor relationships. CHRs also recognized the benefits of COPE trainings in health topics and Motivational Interviewing, as well as the flipcharts that were widely used. Overall, CHRs perceived that COPE’s programmatic support has strengthened their validity and reputation with providers and clients as well as enhanced their ability to positively effect health outcomes among their clients. Though we hoped to compare the results of the implementation of COPE to other programs, a literature review matched on-site stakeholder feedback that there are no programs similar to COPE to compare across published program results.

Recognizing the importance of “clinic-community linkages,” we were particularly interested in effecting change through stronger ties between Community Health Workers and clinic-based providers [16, 17]. Our findings suggest that COPE has had a positive effect on improving integration between clinical providers and CHRs through closer communication and coordination of care between clinic- and community-based providers. In particular, both qualitative and quantitative findings suggest that the ability of CHRs to access the Electronic Health Record to document their encounters and obtain clinical information on their clients is an important factor for establishing stronger clinic-community linkages. Nonetheless, the CHR experience of these programmatic efforts suggest that further work is needed, particularly to integrate care teams across the continuum of clinic- and community-based providers. Implementing access to EHR and inter-professional case management care teams across all service units could further support this need.

## Conclusion

COPE will continue to work with stakeholders to respond to this data by implementing programmatic changes to address continued needs voiced by the CHRs. Evaluating COPE from provider and client perspectives, including clinical and client-reported outcomes, will also be needed to understand the full effect of this program. COPE may provide a useful programmatic model on how best to support community health workers through strengthened clinic-community linkages, standardized competencies, training support, and structured home-based interventions for high-risk individuals.

## Abbreviations

CAG: COPE advisory group; comprised of health professionals and allied fields in the Navajo Nation
CHAP: Community Health Advisory Panel; comprised of CHRs and their COPE patients in the Navajo Nation
CHR: Community Health Representative (Navajo Nation)
CHW: Community Health Worker
COPE: Community Outreach and Patient Empowerment Program (Navajo Nation)
DM: Diabetes Mellitus
EHR: Electronic Health Records
PHN: Public Health Nurse
